# A method to simulate incentives for cost containment under various cost sharing designs: an application to a first-euro deductible and a doughnut hole

**DOI:** 10.1007/s10198-016-0843-9

**Published:** 2016-11-14

**Authors:** D. Cattel, R. C. van Kleef, R. C. J. A. van Vliet

**Affiliations:** Institute of Health Policy and Management, Erasmus University Rotterdam, P.O. Box 1738, 3000 DR Rotterdam, The Netherlands

**Keywords:** Health insurance, Moral hazard, Deductibles, Cost containment, Expected health expenses, Incentives, I10 Health, G22 Insurance, insurance companies, actuarial studies, D84 Expectations, speculations, C10 General econometric statistical methods and methodology

## Abstract

Many health insurance schemes include deductibles to provide consumers with cost containment incentives (CCI) and to counteract moral hazard. Policymakers are faced with choices on the implementation of a specific cost sharing design. One of the guiding principles in this decision process could be which design leads to the strongest CCI. Despite the vast amount of literature on the effects of cost sharing, the relative effects of specific cost sharing designs—e.g., a traditional deductible versus a doughnut hole—will mostly be absent for a certain context. This papers aims at developing a simulation model to approximate the relative effects of different deductible modalities on the CCI. We argue that the CCI depends on the probability that healthcare expenses end up in the deductible range and the expected healthcare expenses given that they end up in the deductible range. Our empirical application shows that different deductible modalities result in different CCIs and that the CCI under a certain modality differs across risk-groups.

## Introduction

There is a vast amount of literature on the effects of consumer cost sharing on moral hazard [1, 13, 22]. The RAND experiment, for example, has shown that a higher level of cost sharing generally results in less moral hazard [12]. It is therefore not surprising that most health insurance schemes include cost sharing arrangements to provide consumers with incentives for cost containment and counteract moral hazard [2, 8, 14, 16, 21]. Policymakers are faced with choices on the implementation of a specific cost sharing design. Should, for example, a first-euro deductible^1^ (i.e., up to the deductible amount, insured are obliged to pay 100% of their healthcare expenses out-of-pocket in the contract period, generally a calendar year) be favored rather than a ‘doughnut hole’ (i.e., insured experience a gap in coverage starting after they have incurred a fixed amount of healthcare expenses)? In this case, policymakers decide on the timing of onset of a deductible during the contract period. Under a first-euro deductible, the timing is initial, while under a ‘doughnut hole’ the timing of onset is delayed, since individual healthcare expenses are required before this modality comes into effect. One of the guiding principles in this decision process on the cost sharing design could be which specific cost sharing design is expected to lead to the strongest incentives for cost containment.

Despite the vast amount of literature on the effects of cost sharing, the relative effects of specific cost sharing designs will mostly be absent. In these situations, methods to simulate incentives for cost containment under various cost sharing designs may be helpful for policymakers to underpin decisions on the design of effective consumer cost sharing in health insurance. To the best of our knowledge, such a method is not yet described in the literature. This paper focuses on the deductible as a cost sharing mechanism and aims at developing a simulation model to approximate the relative effects of different deductible modalities on incentives for cost containment. We simulate the individual’s cost containment incentives (henceforth referred to as the CCI) as expected at the start of the contract period, given the individual’s expected healthcare expenses. We focus solely on the CCI at the start of the insurance contract—rather than on the evolution of the CCI during the contract period—since benefit design decisions are usually made prior to the start of the insurance contract. In addition to developing a simulation method, we empirically illustrate this method for a first-euro deductible and a doughnut hole.^2^ In this illustration we will simulate average CCIs for the total population and, separately, CCIs for groups of low-risk individuals and high-risk individuals.

Our method is based on the classical economic theory that consumers act like a homo economicus and possess traits such as perfect self-interest, rationality, and information. For the homo economicus the CCI is affected by the marginal out-of-pocket expenses given the individual’s expected spending in the contract period. We will argue that these marginal out-of-pocket expenses depend on two parameters. The first parameter is the probability that individual healthcare expenses end up in the deductible range. Ceteris paribus, the CCI is expected to decrease with this probability. The explanation is that individuals will hardly experience any incentives for cost-conscious behavior when they expect their expenses to (far) exceed the deductible range; any savings will reduce the insurance claim, but not their out-of-pocket expenses [10, 12]. Given that expenses of an individual end up in the deductible range (hypothetically speaking), there is a second parameter of concern: the total expected expenses in the deductible range.^3^ The higher the total expected expenses—given that they end up in the deductible range—the higher the savings potential is, and the stronger the CCI will be.

The structure of this paper is as follows. In the next section, the two deductible modalities under study are introduced followed by a section in which the relevant parameters for approximating the CCI are specified. The subsequent section briefs about the conceptual framework to simulate the CCI. Data and methods are described in the following two sections. Results are presented before the concluding section. Finally, conclusion and discussion are summarized.

## Deductible modalities

In our conceptual model and empirical illustration we study two deductible modalities applied in practice: (1) a first-euro deductible and (2) a doughnut hole.

**Fig. 1:**
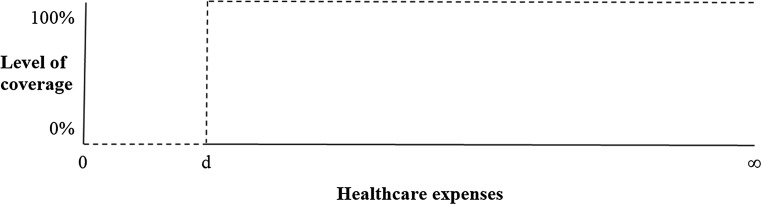
Insurance under a first-euro deductible with range [0, *d*]

A first-euro deductible is the most commonly applied deductible modality and implies that patients pay the first €*d* of healthcare expenses out of their own pocket, before the insurer takes over and reimburses all excess healthcare expenses covered by the benefit package. The timing of onset of this deductible is initial. In Fig. 1 expenses in the interval [0, *d*] are borne by the insured, while expenses in the interval [*d*, ∞] are borne by the insurer. First-euro deductibles can be, for example, found in the US, the Netherlands and Switzerland.

**Fig. 2:**
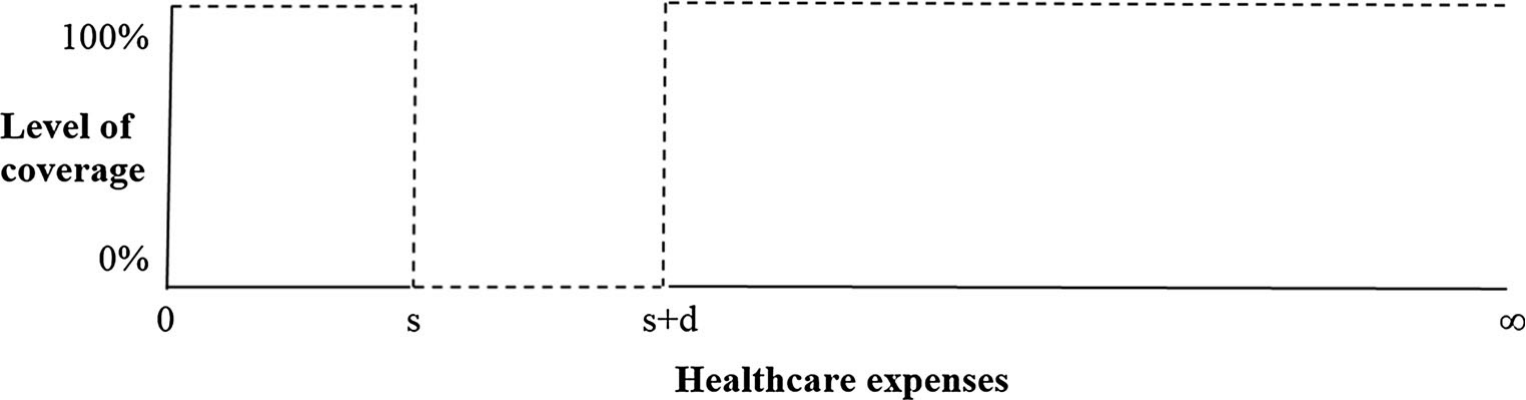
Insurance under a doughnut hole with range [*s*, *s* + *d*]

A doughnut hole is a deductible that starts at a higher level of healthcare expenses than €0. In contrast to a first-euro deductible, the timing of onset of this deductible modality is delayed, since individual healthcare expenses are required before this modality comes into effect. A ‘doughnut hole’ can be seen as a ‘shifted’ deductible with a uniform starting point. The latter means that the starting point of the doughnut hole is fixed for all individuals and set, for example, at the mean of actual healthcare expenses in the population in the previous year. Figure 2 shows that full coverage is provided for those expenses ranging from 0 to the starting point of the doughnut hole (interval [0, s]). Then, insured enter the doughnut hole and experience a gap in coverage. Healthcare expenses from the starting point of the deductible *s* until the endpoint *s* + *d* must be paid out-of-pocket (interval [*s*, *s* + *d*]). Full coverage is again provided by the insurer if healthcare expenses exceed the doughnut hole (interval [*s* + *d*, ∞]). An example of this modality can be found in the Medicare drug coverage system that was implemented in 2006 in the US (part D).

## Incentives for cost containment: What are the relevant parameters?

Our framework starts from the idea that consumers behave rationally. Though this assumption is probably unrealistic and over-simplistic, it provides a theoretical starting point for the development of our framework. As we will discuss in the end of this paper, we believe it is possible to extend the framework with other assumptions on consumer behavior that may follow from (future) empirical studies. The central point of our framework is that for a perfectly rational consumer the CCI in a deductible plan depends on the marginal out-of-pocket expenses given the expected spending in the contract period. More specifically, we will argue that the CCI depends on (1) the probability that individual healthcare expenses end up in the relevant deductible range and (2) the total expected expenses given that they end up in the relevant deductible range. The relevant deductible range represents the interval where the individual, instead of the insurer, bears the costs. In the following two subsections we discuss these two parameters in more depth.

### The probability that individual healthcare expenses end up in the relevant deductible range

**Fig. 3:**
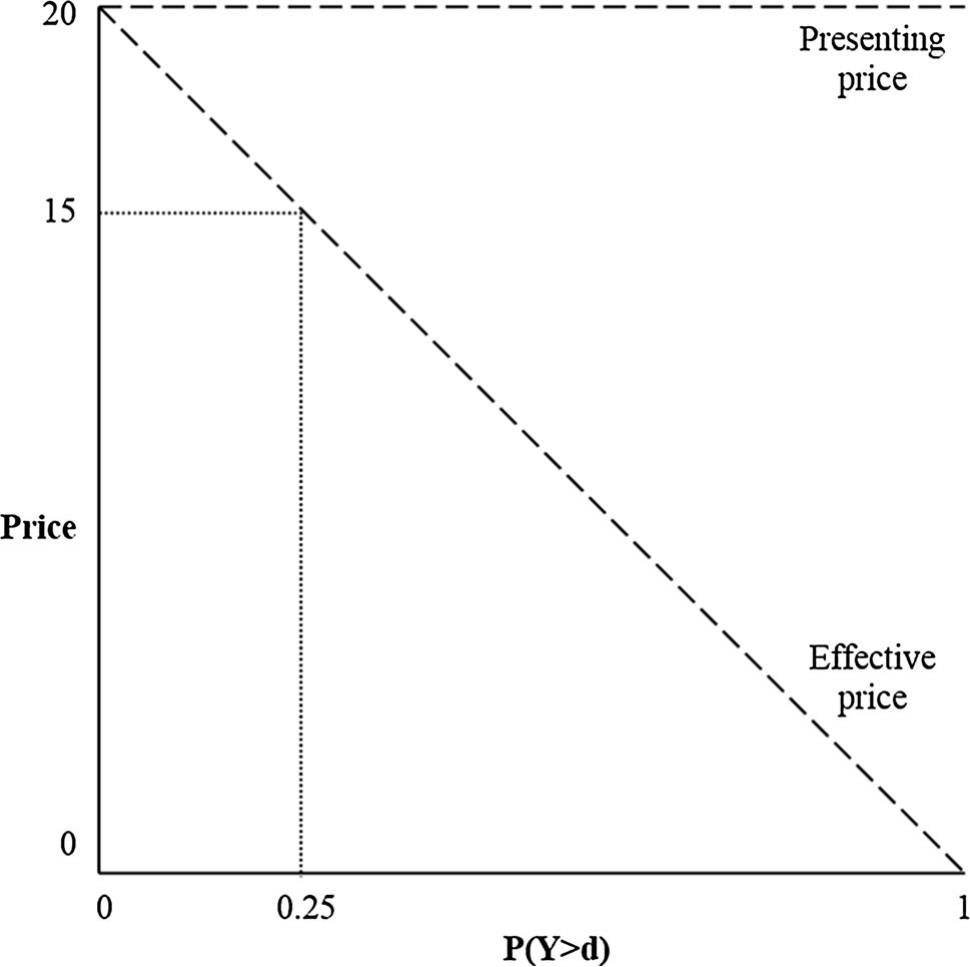
Presenting price versus effective price under a deductible (*P* probability, *Y* healthcare expenses, *d* deductible amount)

Theory predicts that, in case of a first-euro deductible, the price sensitivity of an individual is negatively correlated with the probability that healthcare expenses exceed the deductible amount, ceteris paribus [10, 12]. For a doughnut hole, the price sensitivity of an individual is expected to be negatively correlated with the probability that healthcare expenses do not fall in the deductible range, keeping other things equal. This principle can be illustrated by the following anecdotal example from Newhouse [14:81]: ‘Consider a consumer on the Experiment plan with a 50% coinsurance plan and a $1000 maximum dollar expenditure (MDE). In any year, this person will have free care after spending $2000 on healthcare services. Suppose the person knows in advance that she will spend at least $2000; then any additional care she decides to purchase today is, in effect, free. Alternatively, suppose the person knows that she will not spend as much as $2000; then any additional care she decides to purchase today will cost 50 cents on the dollar because she will not anticipate free care later in the year.’ This example implies that a utility-maximizing consumer uses the presenting price of a visit (i.e., the real price) minus the product of the probability to exceed the MDE and the presenting price to determine whether a visit is worth its costs. This can be defined as the effective price [12]. For example, if the probability of exceeding the deductible amount is 0.25, the effective price for healthcare to the insured of a €20 visit is €15 (€20 minus the product of 0.25 and €20). The principle of varying effective prices with the probability of having ‘free’ healthcare is shown in Fig. 3.

The theory of effective prices suggests that, in some cases, an individual perceives himself as completely insured or completely uninsured and thus experiences a weak or strong CCI. For example, if for a first-euro deductible the probability that healthcare expenses exceed the deductible amount approximates 0, the individual perceives himself as completely uninsured and the effective price equals the presenting price, which suggests a relatively strong CCI. In contrast, if for a first-euro deductible the probability that healthcare expenses exceed deductible amount is close to 1, the individual perceives himself as completely insured and the effective price is €0 which implies a relatively weak CCI. In the latter case, cost-conscious behavior will not prevent the individual from reaching the maximum on out-of-pocket expenses [12, 17, 18]. Under a first-euro deductible, an individual thus perceives himself as completely uninsured if he knows for sure—hypothetically speaking—that total healthcare expenses end up in the interval [0, *d*]. Under a doughnut hole, this is the case if an individual knows for sure that total healthcare expenses end up in the doughnut hole (interval [*s*, *s* + *d*]). In contrast, an individual perceives himself as completely insured under a first-euro deductible, if he knows for sure that total healthcare expenses will end up in the interval [*d*, ∞]. Under a doughnut hole, this is the case if the individual knows for sure that total expenses end up in the intervals [0, *s*] or [*s* + *d*, ∞]. Though it is unrealistic to assume that individuals know for sure whether or not healthcare expenses end up in a specific deductible interval, the aforementioned examples illustrate how the CCI depends on the probability to end up in the deductible range.

Theoretically, the probability that an individual’s healthcare expenses end up in the deductible range depends on three parameters: (1) the amount of healthcare that is already used in the contract period, (2) the number of days remaining in the contract period, and (3) the expected healthcare expenses for the remainder of the contract period [10]. Since we focus on the CCI at the start of the contract period (and not on how the CCI evolves through the contract) the first two parameters are not relevant here.^4^ This implies we will solely focus on the link between expected spending and the CCI. In general, higher expected spending at the start of the contract period implies a higher probability to exceed the deductible.

### The total expected expenses given that they end up in the relevant deductible range

As discussed in the previous subsection, the probability that healthcare expenses end up in the deductible range is an important determinant in approximating the CCI. Nevertheless, we argue it is not the only relevant parameter. Consider the following hypothetical situation where two individuals are subject to a first-euro deductible of €500. Both individuals know with certainty that healthcare expenses remain below this deductible amount.^5^ Assume that person A has expected expenses in the deductible range of €100 and person B has expected expenses in the deductible range of €400. In this case, it would be inaccurate to conclude that the CCIs for these individuals are equal. In this specific case, B has a stronger CCI than A, since the expected expenses for which the individual is price sensitive due to the probability of not exceeding the deductible are higher for B than for A. In other words, B has a higher savings potential than A. Building on this example, we state that the expected healthcare expenses given that they end up in the deductible range is a relevant parameter for the CCI too.

## A method to simulate incentives for cost containment

In this section we build a conceptual framework to simulate the CCI under different deductible modalities at the start of the contract period. We describe our method for a first-euro deductible and a doughnut hole.

### First-euro deductible

Under a first-euro deductible, the deductible range where the individual bears the costs equals [0, *d*]. Accordingly, the CCI under a first-euro deductible can be simulated by combining the probability *P* that individual healthcare expenses *Y* remain below the deductible amount *d* and the expected expenses *E*(*Y*) given that expenses *Y* remain below the deductible amount *d*:

1 $$ {\text{CCI}}_{{{\text{first}} - {\text{euro\,deductible}}}} = P\left( {Y < d} \right)*E(Y|Y < d). $$

**Fig. 4:**
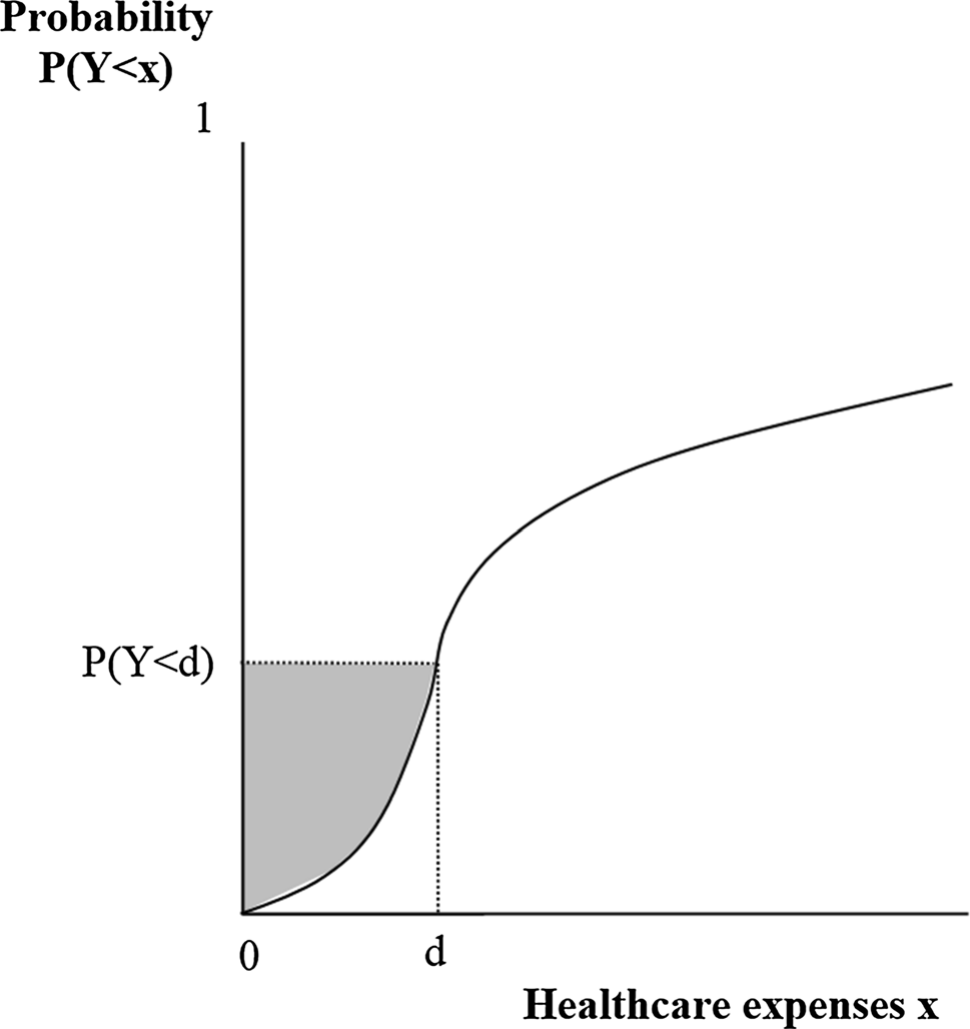
CCI under a first-euro deductible

The essence of the CCI can be graphically illustrated with Fig. 4. Consider the curve in Fig. 4 to represent the probability of an individual’s healthcare expenses to remain below amount *x*. For an infinite value of *x*, this probability equals 1, which means that all expenses are in the interval [0, *x*]. In this extreme case *E*(*Y|Y* < *x*) equals *E*(*Y*) and the outcome of Eq. (1) exactly represents the total area above the curve. This is no longer true, however, when *P*(*Y* < *x*) is smaller than 1, which is the case for *x* = *d*. Since *P*(*Y* < *d*) is smaller than 1 and *E*(*Y|Y* < *d*) is smaller than *E*(*Y*), the outcome of Eq. (1) no longer represents the total area above the curve, but shrinks to the shaded area. Here we come to the essence of our method: when the shaded area of deductible modality A is larger than that of deductible modality B, the CCI is expected to be stronger under modality A than under modality B.

### Doughnut hole

Under a doughnut hole, the endpoint of the deductible range is marked by *s* + *d*. *P*(*Y* < *s* + *d*) and *E*(*Y|Y* < *s* + *d*) are higher compared to *P*(*Y* < *d*) and *E*(*Y|Y* < *d*) under a first-euro deductible with deductible amount *d*. Consequently, the CCI for the interval [0, *s* + *d*] will be stronger than the CCI for the interval [0, *d*]. It is incorrect, however, to assume that the CCI under a doughnut hole equals the CCI for the complete interval [0, *s* + *d*]. This can be illustrated with an infinite value for *s*: here both *P*(*Y* < *s* + *d*) and *P*(*Y* < *s*) equal 1. In this case it would be inaccurate to conclude that the CCI equals *P*(*Y* < *s* + *d*) * *E*(*Y|Y* < *s* + *d*), since all expenses are in the interval [0, *s*] and are fully reimbursed by the insurer. In other words, no expenses appear in the interval [*s*, *s* + *d*] where the individual bears the costs. So, we argue that, in this specific example, the CCI should equal 0 and, in general, the negative effect of interval [0, *s*] on the CCI should be incorporated in the calculation of the CCI. The latter implies that when determining the CCI under a doughnut hole, the focus should be on the expenses where the insured are price sensitive due to the probability of entering the doughnut hole but not reaching the endpoint of the doughnut hole.

This reasoning implies that the CCI under a doughnut hole can be approximated by the product of *P*(*Y* < *s* + *d*) and *E*(*Y|Y* < *s* + *d*) minus the product of *P*(*Y* < *s*) and *E*(*Y|Y* < *s*). Accordingly, the CCI under a doughnut hole can be calculated by Eq. (2).

2 $$ {\text{CCI}}_{\text{doughnut\,hole}} = \left[ {P\left( {Y < s + d} \right)*E\left( {Y|Y < s + d} \right)} \right] - \, \left[ {P\left( {Y < s} \right) * E\left( {Y|Y < s} \right)} \right]. $$

**Fig. 5:**
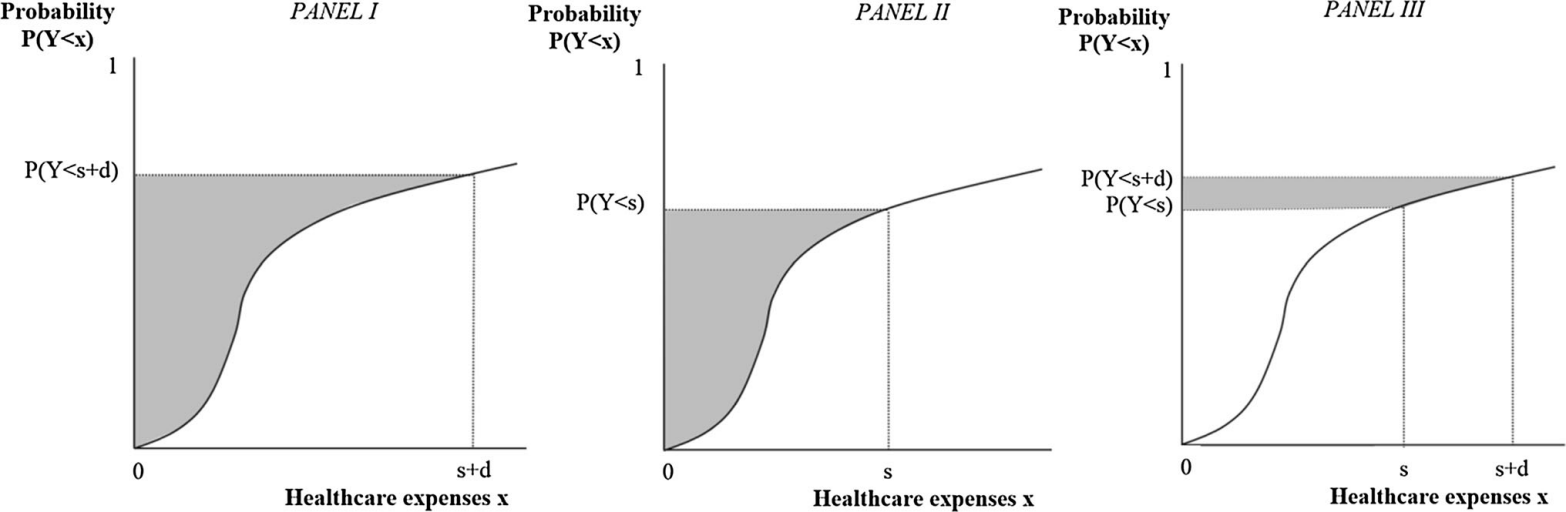
The CCI under a doughnut hole

This procedure is graphically illustrated in Fig. 5 where the shaded area in panel I represents *P*(*Y* < *s* + *d*) * *E*(*Y|Y* < *s* + *d*), the shaded area in panel II represents *P*(*Y* < *s*) * *E*(*Y|Y* < *s*), and the shaded area in panel III represents the outcome of Eq. (2).

## Data

For the empirical application of our method we used administrative data from Dutch insurers operating under the Health Insurance Act. We used a sample of 500,000 individuals who were randomly selected from the total Dutch population of 18 years and older and enrolled in the basic health insurance for a complete calendar year (2011). The sample is similar to the total Dutch population regarding mean, standard deviation, minimum, and maximum.

The dataset includes individual-level risk-information on healthcare expenses and risk-characteristics. The risk-characteristics are age-gender classes, diagnoses cost groups (DCGs), pharmacy-based cost groups (PCGs), high cost groups (HCGs) and multiple prior years high costs (MHCs). In the Netherlands this information is used in the Dutch risk-equalization system. Further information on these risk characteristics can be found in previous work (see, for example, [19]). In addition to information on risk-characteristics, the dataset includes information on total healthcare expenses in 2011 that are covered by the Dutch basic health insurance (e.g., costs for general practitioner care, hospital care, pharmaceutical care and mental care). Based on visual inspection, we excluded 10 insured with extremely high healthcare expenses ranging from €223,184 till €467,722 from the full sample of 500,000 insured, because they appeared to negatively affect our expenditure model. On average in the selected sample of 499,990 individuals, the actual healthcare expenses were €2257 with a standard deviation of €6124, a minimum of €1, a median of €593 and a maximum of €217,566.

## Methods

To empirically illustrate our method for simulating the CCI under different deductible modalities we follow a four-step procedure:

1. Estimate an expenditure model;
2. Approximate the probability that healthcare expenses end up in the deductible range;
3. Approximate the expected expenses given that they end up in the deductible range;
4. Simulate the CCI.

In this paper we are interested in the CCI under a specific deductible modality relative to others; absolute figures of the CCI are of little significance. Empirical results are intended as an illustration of the method developed. First, we derive the CCI under a first-euro deductible of €500, €1000, €2000, €3000, €4000, €5000 and €10,000 in order to examine the effects of the deductible amount. After that, we examine the CCI under a doughnut hole of €1000 with a uniform starting point at €500, €1000, €2000, €2257 (i.e., the mean of actual healthcare expenses in the selected sample of 499,990 individuals), €3000, €4000 and €5000 in order to compare the CCI between a first-euro deductible and a doughnut hole. Average CCIs under the two deductible modalities are simulated for the full sample, and separately, for a group of high-risk individuals and the complementary group of low-risk individuals. Morbidity information is used to determine to which risk-group an individual belongs: those individuals with (without) a DCG, PCG, HCG and/or MHC are considered as a high-risk individual (low-risk individual). In this sample 72% is considered as a low-risk individual and 28% as a high-risk individual.

It is important to mention that—next to the assumption on rational behavior—our concept is based on some other (implicit) assumptions. For example, we assume a linear relationship between the probability that healthcare expenses end up in the deductible range and the CCI. Furthermore, we focus on the CCI regarding total healthcare utilization that is subject to the deductible and neglect the composition of the care that is used. The implications of these and other assumptions, will be discussed in the last section of this paper.

### Estimate an expenditure model

First, to predict expected healthcare expenses *E*(*Y*) for each individual, an expenditure model is estimated with actual expenses in 2011 as dependent variable and age-gender classes, DCGs, PCGs, HCGs and MHCs as explanatory variables. We opted for a Generalized Linear Model (GLM) with a gamma distribution and a log-link function, which is considered to be an appropriate statistical method for modelling healthcare expenses in many studies (see, for example, [3, 5, 7, 11, 17]). Basically, all risk characteristics are statistically significant at the conventional level (given the large sample size). On average the expected healthcare expenses were €2537 with a standard deviation of €7762, and the *R* ^2^ of the model is 0.39. In the subsequent tables we show that our estimation approach provides an acceptable fit between the actual and predicted parameters of the CCI.^6^

### Approximate the probabilities that healthcare expenses end up in the deductible range

After estimating an expenditure model, the probability *P* that healthcare expenses *Y* remain below deductible amount *d*, starting point *s* and endpoint *s* + *d* is approximated. We follow the procedure as described by van Kleef and colleagues [17], who have identified the relevant parameters given the use of a gamma distribution with a log-link. The probabilities that we are interested in can be derived by Eqs. (3) till (5).

3 $$ P\left( {Y < d} \right) = \varGamma \left( {c_{d} ,k} \right), $$

4 $$ P\left( {Y < s} \right) = \varGamma \left( {c_{s} ,k} \right), $$

5 $$ P\left( {Y < s + d} \right) = \varGamma \left( {c_{s + d} ,k} \right), $$

where *Γ*(.) is the cumulative density function of the gamma distribution, the scale parameter *k* is 0.4969, and:

6 $$ \lambda = k / E(Y), $$

7 $$ c_{d} = d*\lambda , $$

8 $$ c_{s} = s*\lambda , $$

9 $$ c_{s + d} = (s + d)*\lambda . $$

Given the assumptions made and given our dataset, we check whether the results based on Formulae (3) till (9) are in line with the actual figures in the sample; the proportion *ρ* and probability *P* that healthcare expenses *Y* remain the deductible amount *d* under a first-euro deductible are compared. Table 1 shows that *ρ*(*Y* < *d*) and *P*(*Y* < *d*) follow the same pattern, specifically in case of a relatively high deductible amount.

**Table 1:** Proportions *ρ* and probabilities *P* that healthcare expenses *Y* remain below various deductible amounts *d* for the full sample

*d*	*ρ*(*Y* < *d*)	*P*(*Y* < *d*)
500	0.47	0.43
1000	0.61	0.57
2000	0.75	0.73
3000	0.82	0.81

### Approximate the expected expenses given that they end up in the deductible range

Given expected expenses *E*(*Y*) and the parameters calculated in the previous step, expected expenses given that expenses end up in the interval [0, *d*], [0, *s*], respectively [0, *s* + *d*] can be calculated by Eqs. (10), (11) and (12) [17].

10 $$ E\left( {Y|Y < d} \right) = E\left( Y \right)*\varGamma \left( {c_{d} ,k + 1} \right) / \varGamma (c_{d} ,k), $$

11 $$ E\left( {Y|Y < s} \right) = E\left( Y \right)*\varGamma \left( {c_{s} ,k + 1} \right) / \varGamma (c_{s} ,k), $$

12 $$ E\left( {Y|Y < s + d} \right) = E\left( Y \right)*\varGamma \left( {c_{s + d} ,k + 1} \right) / \varGamma (c_{s + d} ,k). $$

Table 2 shows the actual expenses and expected expenses given that expenses remain below first-euro deductible amount *d.* Our approach somewhat underestimates these expenses for the relatively small first-euro deductibles and somewhat overestimates them for the higher ones, but these deviations do not seem important.

**Table 2:** Mean of actual expenses *Y* and expected expenses *E*(*Y*) given that expenses *Y* remain below various deductible amounts *d* for the full sample

*d*	*Y*|*Y* < *d*	*E*(*Y*|*Y* < *d*)
500	186	158
1000	314	302
2000	517	551
3000	688	755

Based on the results presented in Tables 1 and 2, there seems to be no reason to believe that the overestimations of the mean and the standard deviation of expected healthcare expenses compared to the actual healthcare expenses have unacceptable effects on the key parameters of interest in this paper.

### Simulate the CCI

As discussed in section “A method to simulate incentives for cost containment”, the CCI is conceptualized as a product of the probability that individual healthcare expenses end up in the deductible range and the expected expenses given that they end up in the deductible range. Therefore, parameters obtained in step 2 and step 3 are combined in order to determine the CCI for each individual. The CCI under a first-euro deductible with deductible amount *d* is calculated by Eq. (1). The CCI under a doughnut hole with starting point *s* and deductible amount *d* is approximated by Eq. (2). The CCI is presented in Euros and can be interpreted as the marginal amount of healthcare expenses for which a consumer is fully price sensitive. Hypothetically speaking, the CCI will be zero for a consumer who knows for sure his spending will exceed the deductible amount. For a consumer who knows for sure his spending will not exceed the deductible amount, the CCI will equal his expected spending.

### Implications

At least three implications arise from the conceptual framework as described in section “A method to simulate incentives for cost containment”. These hypotheses are to be addressed in “Results” where the simulation results are presented. First, the CCI under a deductible increases when the deductible amount increases. If, ceteris paribus, the deductible amount increases (i.e., point *d* and, accordingly, point *s* + *d* is shifted to the right), the deductible range is broadened. As a result, both the probability that expenses end up in the deductible range and the expected expenses in the deductible range once they ended up in the deductible interval are expected to increase. This will result in a stronger CCI.

Second, we expect that different deductible modalities lead to different CCIs. Shifting the deductible influences the CCI. The direction of the effect is an interesting empirical question. On the one hand, a shift of the deductible to higher expenditure levels reduces the probability to reach the deductible range, which negatively affects the CCI. On the other hand, such a shift increases the expected expenses given that they end up in this range, which positively affects the CCI.

**Fig. 6:**
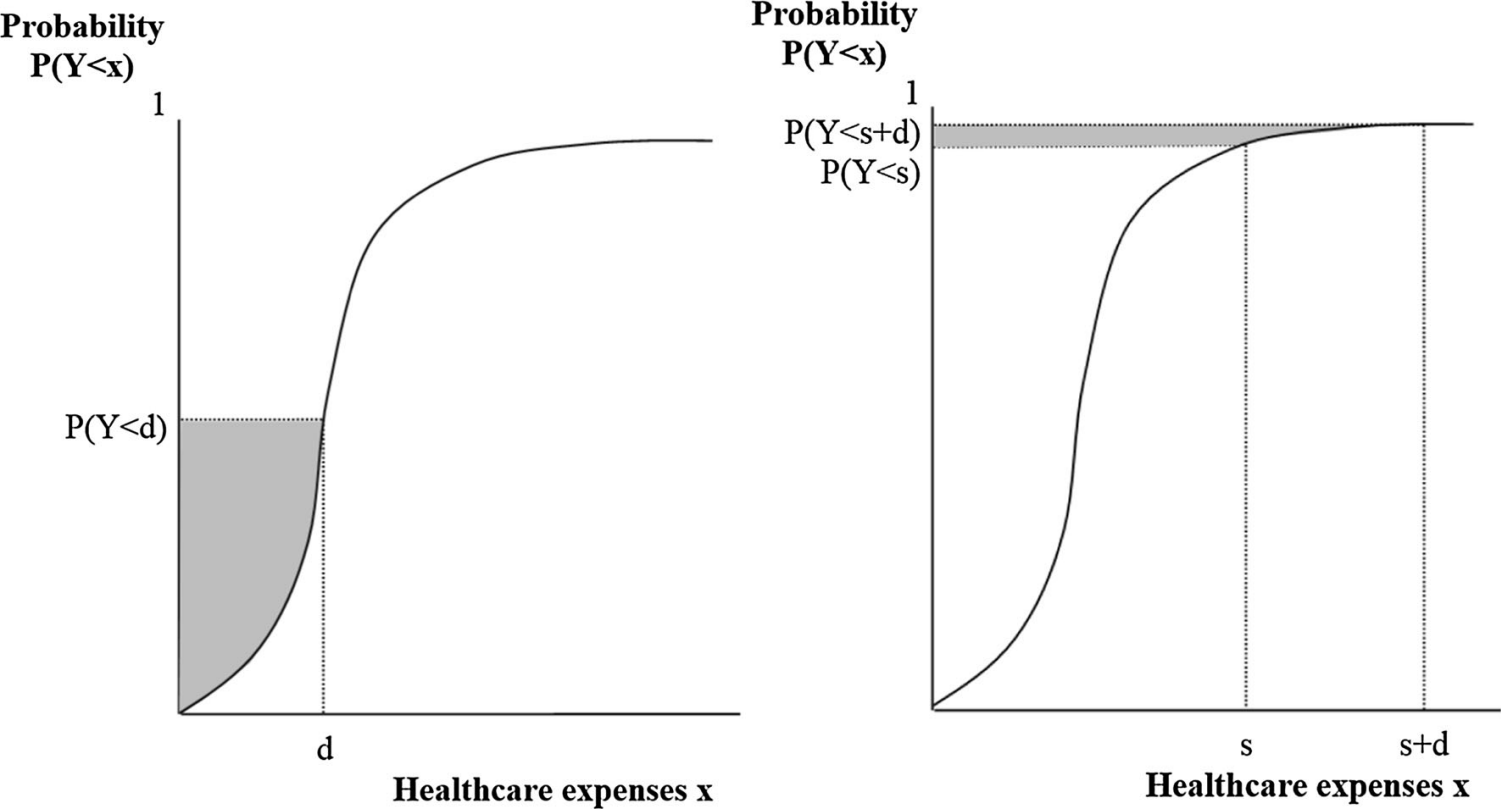
The CCI for a low-risk individual under a first-euro deductible (*left panel*) and under a doughnut hole with a starting point at the mean of actual healthcare expenses in the population (*right panel*)

Third, we hypothesize that the CCI under a first-euro deductible and a doughnut hole will differ across risk-groups. Figure 6 shows *P*(*Y* < *x*) of a relatively low-risk individual under a first-euro deductible (left panel) and under a doughnut hole with a starting point at the mean of actual healthcare expenses in the population (right panel). *E*(*Y*) for this healthy individual are relatively low, but there is always a certain level of uncertainty whether or not this individual needs care. This implies that, under a first-euro deductible, there is a low probability that healthcare expenses exceed the deductible amount. In contrast, under a doughnut hole with a starting point at the mean of healthcare expenses, it is not very likely that this low-risk individual ends up in the doughnut hole. *P*(*Y* < *s*) and *P*(*Y* < *s* + *d*) both approximate 1. As a result of the relatively high *P*(*Y* < *d*) under a first-euro deductible compared to *P*(*s* < *Y* < *s* + *d*) under a doughnut hole, the CCI for this low-risk individual is relatively strong in case of a first-euro deductible in comparison to a doughnut hole.

**Fig. 7:**
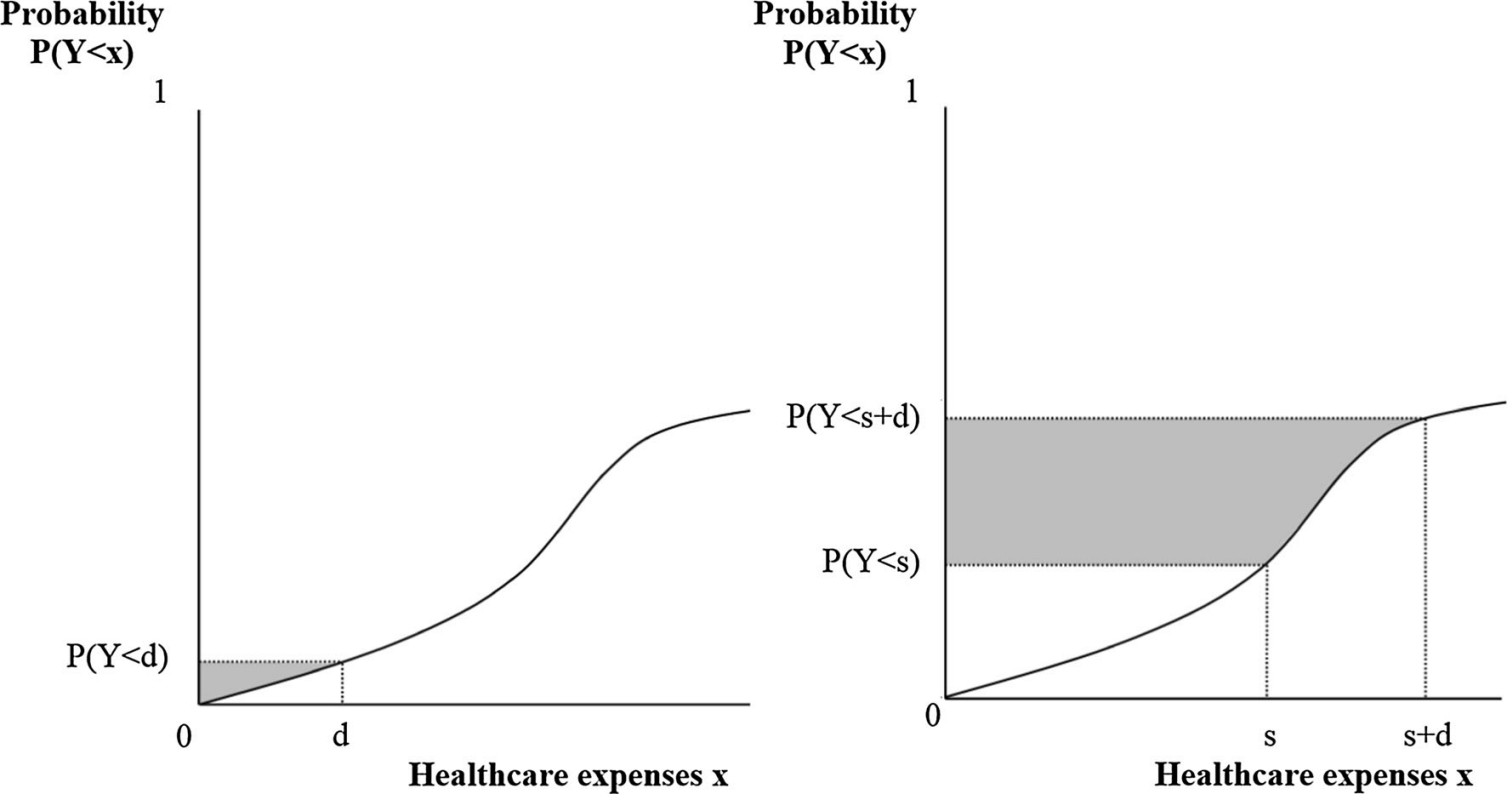
The CCI for a high-risk individual under a first-euro deductible (*left panel*) and under a doughnut hole with a starting point at the mean of actual healthcare expenses (*right panel*)

Now consider a relatively high-risk individual, such as a chronically ill patient. *P*(*Y* < *x*) is depicted in Fig. 7. *E*(*Y*) for this relatively unhealthy individual are above average. Accordingly, under a first-euro deductible, *P*(*Y* < *d*) is low (Fig. 7, left panel). In contrast, *P*(*s* < *Y* < *s* + *d*) is relatively high when the starting point of the doughnut hole is set at the mean of actual healthcare expenses (Fig. 7, right panel). Consequently, for this high-risk individual the CCI is relatively strong in case of a doughnut hole in comparison to a first-euro deductible.

The previous consideration implies that, at the population level, it is not obvious whether a first-euro deductible leads to a stronger or weaker CCI than a doughnut hole. On the one hand, a shift of the starting point of the deductible to a higher expenditure level than €0 may increase the CCI for the high-risk individuals (a relatively small group with relatively high savings potential). On the other hand, such a shift may decrease the CCI for the low-risk individuals (a relatively large group with relatively low savings potential). In our empirical illustration we aim to simulate the net outcome of these two effects.

## Results

As an illustration of the method developed, this section presents the empirical results for a first-euro deductible and a doughnut hole. Results are shown for the full sample and also separately for a group of high-risk individuals and the complementary group of low-risk individuals.

### Full sample

In Table 3 the results are presented for a first-euro deductible of various deductible amounts for the total sample. The mean probability that healthcare expenses remain below the deductible amount, the expected expenses given that they remain below the deductible amount, and the product of these two parameters are shown. As hypothesized in subsection “Implications”, Table 3 reveals that an increase in the deductible amount indeed leads to a higher *P*(*Y* < *d*) and higher *E*(*Y|Y* < *d*). Thus, the higher the deductible amount is, the stronger the CCI will be. Note that this conclusion also holds for a doughnut hole, as *P*(*s* < *Y* < *s* + *d*) and *E*(*Y|s* < *Y* < *s* + *d*) increase with a higher deductible amount.

**Table 3:** The CCI under a first-euro deductible of various deductible amounts *d* for the full sample

*d*	*P*(*Y* < *d*)^a^	E(*Y*|*Y* < *d*)^b^	CCI
500	0.43	158	68
1000	0.57	302	171
2000	0.73	551	393
3000	0.81	755	598
4000	0.86	921	773
5000	0.89	1059	920
10,000	0.96	1475	1371

^a^The probability that healthcare expenses remain below the deductible amount

^b^The expected expenses given that they remain below the deductible amount

Table 4 shows (the relevant parameters for determining) the CCI under a doughnut hole of €1000 with various starting points (the CCI under a doughnut hole assuming other deductible amounts is shown in the Appendix). The mean probability that healthcare expenses remain below the starting point, respectively the endpoint of the deductible, the expected expenses given that they end up in the interval [0, *s*], respectively [0, *s* + *d*] and the CCI are shown. Table 4 shows that *P*(*Y* < *s*) is lower compared to *P*(*Y* < *s* + *d*). Similarly, *E*(*Y|Y* < *s*) are lower compared to *E*(*Y|Y* < *s* + *d*). Second, results suggest that the CCI under a doughnut hole with deductible amount €1000 increases when the starting point of the doughnut hole is shifted to the right until a starting point of €1000 is used. On average, a stronger CCI is realized under a doughnut hole with a starting point at €1000 compared to a starting point at the mean of actual healthcare expenses in the sample (i.e., 2257). These results imply that, given the dataset and the assumptions made, the ‘sweet spot’ of the starting point is located somewhere around €1000. This finding might suggest that the starting point of the doughnut hole should be located *below* the overall mean of actual healthcare expenses, implying that the starting point of the doughnut hole in the Medicare drug coverage system should be lowered, since it is currently set at the overall mean of actual healthcare expenses.

**Table 4:** The CCI under a doughnut hole with deductible amount *d* €1000 with various starting points *s* for the full sample

*s*	*P*(*Y* < *s*)^a^	*P*(*Y* < *s* + *d*)^b^	*E*(*Y*|*Y* < *s*)^c^	*E*(*Y*|*Y* < *s* + *d*)^d^	CCI
0^e^	0	0.57	0	302	171
500	0.43	0.66	158	433	215
1000	0.57	0.73	302	551	222
2000	0.73	0.81	551	755	204
2257	0.75	0.82	607	801	197
3000	0.81	0.86	755	921	175
4000	0.86	0.89	921	1059	147
5000	0.89	0.92	1058	1173	123

^a^The probability that healthcare expenses remain below the starting point of the deductible

^b^The probability that healthcare expenses remain below the endpoint of the deductible

^c^The expected expenses given that they end up in the interval [0, *s*]

^d^The expected expenses given that they end up in the interval [0, *s* + *d*]

^e^A doughnut hole with a starting point of €0 is effectively a first-euro deductible; the CCI and related probabilities and expected expenses are identical (see Table 3)

A comparison of the results under a first-euro deductible with those under a doughnut hole suggests that different deductible modalities lead to differences in CCIs. Assuming a deductible amount of €1000, a doughnut hole with a relatively low starting point leads on average to a stronger CCI compared to a first-euro deductible. For example, a first-euro deductible of €1000 leads to a CCI of €171 while a doughnut hole of €1000 with a starting point at €1000, respectively at the mean of actual healthcare expenses leads to a CCI of €222, respectively €197. Results suggest that this pattern in favor of a doughnut hole reverses (and the CCI will be stronger in case of a first-euro deductible) when the starting point of the doughnut hole is located somewhere between €3000 and €4000.

### Low-risk individuals and high-risk individuals

Table 5 provides the CCI under a first-euro deductible specifically for the low-risk individuals and the high-risk individuals. For the high-risk individuals *P*(*Y* < *d*) is lower while *E*(*Y|Y* < *d*) are higher in comparison to the low-risk individuals. Under a first-euro deductible, the CCI is strongest for the low-risk individuals compared to the high-risk individuals, as long as the deductible amount is relatively low; when the deductible amount is set somewhere between €4000 and €5000, this pattern is reversed.

**Table 5:** The CCI under a first-euro deductible of various deductible amounts *d* for the low-risk individuals and the high risk individuals

*d*	*P*(*Y* < *d*)^a^	*E*(*Y*|*Y* < *d*)^b^	CCI
*Low-risk individuals*			
500	0.48	157	75
1000	0.63	296	187
2000	0.79	529	418
3000	0.88	709	617
4000	0.92	846	776
5000	0.95	952	899
10,000	0.99	1199	1188
*High-risk individuals*			
500	0.30	162	48
1000	0.41	318	130
2000	0.55	609	329
3000	0.64	875	547
4000	0.70	1119	765
5000	0.75	1341	976
10,000	0.87	2203	1853

^a^The probability that healthcare expenses remain below the deductible amount

^b^The expected expenses given that they remain below the deductible amount

The CCI under a doughnut hole of €1000 with various starting points is shown in Table 6 for the two risk-groups. The CCI under a doughnut hole is stronger for the high-risk individuals than for the low-risk individuals, as long as the starting point of the deductible is shifted to the right considerably. If the starting point is set at a relatively low point (i.e., at €500 or at €1000), the CCI under a doughnut hole is stronger for the low-risk individuals. For the low-risk individuals, the ‘sweet spot’ of the starting point seems to be located somewhere around €1000 while for the high-risk individuals this is somewhere around the overall mean of actual healthcare expenses.

**Table 6:** The CCI under a doughnut hole with deductible amount *d* €1000 with various starting points *s* for the low-risk individuals and the high risk individuals

*s*	*P*(*Y* < *s*)^a^	*P*(*Y* < *s* + *d*)^b^	*E*(*Y*|*Y* < *s*)^c^	*E*(*Y*|*Y* < *s* + *d*)^d^	CCI
*Low-risk individuals*					
0^e^	0	0.63	0	296	187
500	0.48	0.73	157	420	230
1000	0.63	0.79	296	529	231
2000	0.79	0.88	529	709	199
2257	0.82	0.89	580	748	189
3000	0.88	0.92	709	846	159
4000	0.92	0.95	846	952	123
5000	0.95	0.97	952	1031	94
*High-risk individuals*					
0^e^	0	0.41	0	318	130
500	0.30	0.49	162	467	177
1000	0.41	0.55	318	609	200
2000	0.55	0.64	609	875	218
2257	0.57	0.65	680	940	219
3000	0.64	0.70	875	1119	218
4000	0.70	0.75	1119	1341	211
5000	0.75	0.78	1341	1545	200

^a^The probability that healthcare expenses remain below the starting point of the deductible

^b^The probability that healthcare expenses remain below the endpoint of the deductible

^c^The expected expenses given that they end up in the interval [0, *s*]

^d^The expected expenses given that they end up in the interval [0, *s* + *d*]

^e^A doughnut hole with a starting point of €0 is effectively a first-euro deductible; the CCI and related probabilities and expected expenses are identical (see Table 5)

A comparison of the CCI under the two deductible modalities shows that, given our dataset and under the assumptions made in this research, for the low-risk individuals, a doughnut hole on average leads to a stronger CCI compared to a first-euro deductible until a starting point of €3000 or more is chosen. For example, the CCI under a doughnut hole with a starting point at €1000 is €231 compared to the CCI of €187 under a first-euro deductible. Nevertheless, only small differences exist when comparing a first-euro deductible to a doughnut hole with a starting point at the mean of actual healthcare expenses; the CCI equals €187 compared to €189. For the high-risk individuals the CCI is noticeably stronger under a doughnut hole compared to a first-euro deductible, even if the starting point is shifted to the right only moderately. The CCI is, for instance, €177 under a doughnut hole with a starting point at €500 compared to €130 under a first-euro deductible. Results suggest that for the high-risk individuals, a doughnut hole with a starting point at the mean of actual expenditures leads to a stronger CCI compared to a first-euro deductible (€219 compared to €130).

## Conclusion and discussion

Starting from the traditional economic theory that consumers act like a homo economicus, this paper has developed a method to simulate Cost Containment Incentives (CCI) under different deductible modalities. For a homo economicus the CCI depends on two parameters: (1) the probability that individual healthcare expenses end up in the deductible range and (2) the total expected healthcare expenses given that they end up in the deductible range. We have empirically illustrated the method for two modalities applied in practice, i.e., a first-euro deductible and a doughnut hole. Given our dataset and under the assumptions made, our findings lead to four conclusions.

First, not surprisingly, the CCI increases with the deductible amount, ceteris paribus. The developed method can be used to simulate the impact of a higher deductible on the CCI. Second, the CCI differs between deductible modalities. Which deductible modality is opted for by policymakers seems to have consequences in terms of the CCI and it can thus be valuable to take the CCI into consideration when comparing the effectiveness of these different deductible designs. In our sample, a doughnut hole with a well-chosen starting point (i.e., below €4000) on average provides a stronger CCI than a first-euro deductible. This would imply that, to realize a strong CCI, the starting point of the deductible should be higher than zero for all insured. This finding is in line with the conclusion of van Kleef and coauthors [17]. Third, the CCI differs across risk-groups. We have found that under a first-euro deductible the CCI is strongest for the low-risk individuals, as long as the deductible amount is relatively low (i.e., until the deductible amount is set somewhere between €4000 and €5000). Under a doughnut hole, the CCI is strongest for the high-risk individuals, as long as the starting point is higher than €1000. Our findings suggest that the CCI is stronger under a doughnut hole than under a first-euro deductible for both the low-risk individuals—at least when a starting point below €3000 is chosen—and for the high-risk individuals. Fourth, our results suggest that, in order to provide a stronger CCI, the starting point of the doughnut hole should not be located at the mean of actual healthcare expenses in the sample, but somewhere below that mean. This finding suggests that the CCI under the doughnut hole in the Medicare drug coverage system could be increased by lowering the starting point.

It is important to note that our empirical findings depend on several assumptions which deserve further elaboration. In addition, many important topics remain for future research. Six of these issues are discussed below. First, a note of caution should be raised against the assumption of individuals behaving completely rationally, since in practice, insured might actually act differently than the classical theory suggests. First, there is empirical evidence that individuals tend to overestimate small probabilities and underestimate large probabilities [9:279, 20]. This may have consequences for the first parameter in our framework (i.e., the probability that healthcare expenses fall in the deductible range). For example: if a low-risk individual under a first-euro deductible would overestimate the probability of becoming ill, this individual’s perceived probability that healthcare expenses remain below the deductible amount decreases, leading to a weaker CCI. Second, Brot-Goldberg et al. [6] show that, in practice, consumer behavior departures from fully rational behavior in that sense that individuals seem to act in a myopic way. In particular, they show that in the decision of using healthcare, individuals are not responsive to the expected marginal end-of-year price but often respond to easier to understand prices such as spot prices or their prior end-of- year marginal price. This evidence suggests that the second parameter of our framework (i.e., the total expected expenses in the deductible range) might be influenced. Although there is growing empirical evidence on alternative assumptions concerning consumer behavior, there is limited research on how these ‘new’ assumptions should be incorporated in economic simulation studies. It is yet unclear how these insights exactly translate into our simulation framework. For instance, it would be interesting to study how our framework could be extended with weights or additional parameters to incorporate new insights.

Second, in this paper a linear relation between the probability of exceeding the deductible and the CCI is assumed. If there are reasons to believe that an alternative relationship is more realistic, it is possible to interchange the assumption of linearity and plug-in any other relationship in the conceptual framework.

Third, the expected healthcare expenses are an important parameter in the approximation of the CCI. The expenditure model based on age-gender classes, DCGs, PCGs, HCGs and MHCs probably predicts expenses less than perfectly. Therefore, obtained results cannot expected to be perfect either. Overestimated expected expenses might explain why—in contrast to what we hypothesized—a doughnut hole instead of a first-euro deductible leads to the strongest CCI for the low-risk individuals. Further research is needed to simulate the CCI with better prediction models. Significantly better predictions can be expected if expenses in previous years are added to the model, since previous expenses proved to be a strong predictor for future expenses, even when the abovementioned predictors are already included [4, 19]. A better prediction model will likely lead to a larger variance in expected expenses and larger differences in the CCI across risk groups.

Fourth, for reasons of simplicity we did not incorporate a correction for the moral hazard effect. In our empirical illustration we apply a substantially higher deductible amount (i.e., €1000) than the amount originally applied in our data (i.e., €170). If the higher deductible amount was implemented in practice this would have led to less moral hazard and thus lower healthcare expenses. An interesting question is whether or not consumers include the ‘moral hazard effect’ in their expectations about future healthcare expenses. If they do (e.g., by expecting lower healthcare expenses in case of a higher deductible amount) this effect should ideally be incorporated in the type of simulations applied in this paper. This would be possible by modifying the healthcare expenses on which the expenditure model is based.

Fifth, different cost sharing designs are expected to have different implications in terms of solidarity. For example, for the high-risk individuals, a first-euro deductible can be considered as socially inequitable (assuming insufficient financial compensation), because these individuals incur, on average, higher out-of-pocket expenses than their healthy counterparts. In addition, for these high-risk individuals, a first-euro deductible can be considered as ineffective in reducing moral hazard, because these individuals know ex-ante that their yearly healthcare expenses will exceed the deductible amount. The relation between different cost sharing designs and solidarity and to what extent a stronger CCI has an effect on moral hazard reduction might benefit from future research.

Sixth, in this paper only two deductible modalities are empirically illustrated. The method developed allows approximation of the CCI under other deductible modalities as well. Examples of other modalities are a doughnut hole with a risk-adjusted starting point and an income-related deductible. Under a doughnut hole with a risk-adjusted starting point (as proposed in the literature by van Kleef et al. [17]), the location of the doughnut hole depends on specific individual risk-characteristics of the insured, such as demographics, diagnostics or prior healthcare utilization. The starting point could be, for example, based on maximized uncertainty in out-of-pocket expenses or on a maximized CCI. It is expected that a doughnut hole with a risk-adjusted starting point leads to a stronger CCI than a first-euro deductible and a uniform doughnut hole. In addition to the possibility to simulate the CCI under other deductible modalities, the method provides the opportunity to determine the CCI under other forms of cost sharing than deductibles, such as co-insurance (i.e., insured are obliged to pay a percentage of the healthcare expenses per service out-of-pocket) or co-payments (i.e., insured are required to pay a predefined amount per service out-of-pocket). This might be an interesting topic for future research.

Last, we acknowledge that the CCI may be regarded as one of the multiple criteria that can be taken into consideration by policymakers when deciding on the design of effective consumer cost sharing in health insurance. Other criteria, such as the practical and political-ideological aspects of different deductible modalities could be relevant as well. For example, an important aspect in the deductible design decision would be the trade-off between a stronger CCI versus transparency and simplicity. Specifically, in a system with a doughnut hole where the starting point of the deductible depends on individual risk-characteristics, the average CCI might be higher compared to a first-euro deductible, but transparency may be worse when the majority of insured does not understand how and why certain starting points are assigned to them. Consequently, acceptance of the deductible system might be in danger. Another issue would be how policymakers will try to level the government’s cash flow. Switching to a deductible system where a relatively strong CCI can be realized, might lead to a reduction in revenues from deductibles due to more cost-conscious behavior. An option to overcome this reduction in revenues would be to increase the deductible amount [15].

Though the results of our empirical illustration should be interpreted with caution, we believe the method developed in this paper to simulate the CCI can be useful to researchers, insurers and policymakers who want to indicate the relative effects of different cost sharing designs on the incentives for cost-conscious behavior.

## Appendix

See Table 7.

**Table 7:** The CCI under a doughnut hole with various deductible amounts *d* and various starting points *s* for the full sample

*d*	*s*	*P*(*Y* *<* *s*)^a^	*P*(*Y* *<* *s* *+* *d*)^b^	*E*(*Y|Y* *<* *s*)^c^	*E*(*Y|Y* *<* *s* *+* *d*)^d^	CCI
500	0	0	0.43	0	158	68
	500	0.43	0.57	158	302	104
	1000	0.57	0.66	302	433	112
	2257	0.75	0.79	607	709	102
	5000	0.89	0.91	1059	1118	64
2000	0	0	0.73	0	551	393
	500	0.43	0.77	158	658	431
	1000	0.57	0.81	302	755	426
	2257	0.75	0.87	607	959	365
	5000	0.89	0.93	1059	1269	226
3000	0	0	0.81	0	755	598
	500	0.43	0.84	158	842	621
	1000	0.57	0.86	302	921	602
	2257	0.75	0.90	607	1090	505
	5000	0.89	0.94	1059	1348	313
5000	0	0	0.89	0	1059	920
	500	0.43	0.91	158	1118	917
	1000	0.57	0.92	302	1173	872
	2257	0.75	0.93	607	1290	722
	5000	0.89	0.96	1059	1475	451

^a^The probability that healthcare expenses remain below the starting point of the deductible

^b^The probability that healthcare expenses remain below the endpoint of the deductible

^c^The expected expenses given that they end up in the interval [0, *s*]

^d^The expected expenses given that they end up in the interval [0, *s* + *d*]

## Footnotes

1 Or a first-dollar deductible.

2 In this paper we do not pursue optimization of the deductible design. We use designs from practice to illustrate the methodology to simulate the CCI. Nevertheless, the framework can be used as a tool to gain insight in the properties of other deductible modalities and compare deductible designs in terms of the CCI.

3 Expected expenses are considered to be the total expected healthcare expenses that fall under the basic benefit package.

4 Nevertheless, the conceptual framework can be refined to facilitate simulation of the CCI during the contract period. By determining the CCI on multiple moments (i.e., by repeating the procedure that is described in this paper), the other two parameters can be taken into account.

5 Or: both individuals have an *equal* probability that healthcare expenses exceed the deductible amount [i.e., *P*(*Y* < *d*) < 1].

6 We also took into consideration other specifications of the model varying in terms of distribution and link-function. We opted for a GLM with a gamma distribution and a log-link function based on a comparison of mean, standard deviation, minimum, maximum and mean absolute predicted error of actual and expected expenses in the sample and per expenditure quintile.
